# Effective automatic detection of anterior cruciate ligament injury using convolutional neural network with two attention mechanism modules

**DOI:** 10.1186/s12880-023-01091-6

**Published:** 2023-09-11

**Authors:** Chen Liang, Xiang Li, Yong Qin, Minglei Li, Yingkai Ma, Ren Wang, Xiangning Xu, Jinping Yu, Songcen Lv, Hao Luo

**Affiliations:** 1 Department of Minimally Invasive Surgery and Sports Medicine, The 2Nd Affiliated Hospital of Harbin Medical University, Harbin, 150001 China; 2 Department of Control Science and Engineering, Harbin Institute of Technology, Harbin, 150001 China

**Keywords:** Anterior cruciate ligament (ACL) injury, Convolutional neural network (CNN), Magnetic resonance imaging (MRI), Artificial intelligence (AI)

## Abstract

**Background:**

To develop a fully automated CNN detection system based on magnetic resonance imaging (MRI) for ACL injury, and to explore the feasibility of CNN for ACL injury detection on MRI images.

**Methods:**

Including 313 patients aged 16 – 65 years old, the raw data are 368 pieces with injured ACL and 100 pieces with intact ACL. By adding flipping, rotation, scaling and other methods to expand the data, the final data set is 630 pieces including 355 pieces of injured ACL and 275 pieces of intact ACL. Using the proposed CNN model with two attention mechanism modules, data sets are trained and tested with fivefold cross-validation.

**Results:**

The performance is evaluated using accuracy, precision, sensitivity, specificity and F1 score of our proposed CNN model, with results of 0.8063, 0.7741, 0.9268, 0.6509 and 0.8436. The average accuracy in the fivefold cross-validation is 0.8064. For our model, the average area under curves (AUC) for detecting injured ACL has results of 0.8886.

**Conclusion:**

We propose an effective and automatic CNN model to detect ACL injury from MRI of human knees. This model can effectively help clinicians diagnose ACL injury, improving diagnostic efficiency and reducing misdiagnosis and missed diagnosis.

## Background

The anterior cruciate ligament (ACL) starts from the anterior medial aspect of the tibial intercondylar ridge, heals at the anterior angle of the lateral meniscus, and is oblique to the posterior superior lateral aspect, with fibers scalloped to the medial aspect of the lateral femoral condyle. The ACL, one of the ligaments connecting the femur to the tibia in the knee joint, is among the most vulnerable ligaments in the knee joint. It can prevent excessive anterior displacement of the tibia and provides knee stability during rotation [1], making an ACL injury the most common knee ligament injury in athletes. After an ACL injury, the ACL cannot easily heal itself owing inadequate blood supply [2], and it is at high risk for additional damage to the meniscus and cartilage. This damage can cause osteoarthritis (OA) of the knee in the long term, resulting in severe knee pain, deformity, and limited motion on the affected side, which can reduce one’s the quality of life and increase the risk of knee replacement as well as the financial burden on patients. Therefore, timely surgical intervention, such as ACL repair or reconstruction, is usually required after an ACL injury to reduce the risk of additional meniscal and cartilage damage and the long-term OA associated with knee instability [3].

Thus, the accurate assessment of an ACL injury is critical for diagnosis and treatment. The diagnosis of an ACL injury relies primarily on clinician examination of the patient (e.g., anterior drawer test, Lachman test [4]) and magnetic resonance imaging (MRI) [5] of the knee, but diagnosis in this manner depends on the clinical experience of the orthopedic clinicians and on the diagnostic experience of radiologists. The amount of experience has a decisive impact on the diagnosis of an ACL injury, and knee MRI also requires ample time for accurate interpretation. Therefore, a new clinical diagnostic aid system to minimize the underdiagnosis and misdiagnosis of ACL injuries.

In recent years, deep learning by artificial intelligence (AI) image analysis has been widely used in medical imaging. Many deep learning target detection systems are based on a convolutional neural network (CNN) because it can reduce the complexity of the whole network and the training parameters, and it can keep data relatively constant in panning, distortion, and scaling. Furthermore, the network structure is easy to train, optimize, and control. Over time, deep learning has made significant progress in the diagnosis of lung diseases [6, 7], breast cancer [8], thyroid tumors [9], skin lesions [10], sarcopenia [11], meniscus tears [12], and other diseases. Because sports injuries such as ligament tears exhibit subtle abnormalities, clinicians cannot rely entirely on MRI images and physical examination to achieve 100% diagnosis accuracy. As it is impractical to perform the "gold standard" diagnostic method, arthroscopy, on every patient, new complementary diagnostic methods are necessary.

The main objective of our study was to develop a fully automated CNN-based MRI detection system for ACL injury identification using arthroscopy as a reference standard, and to explore the feasibility of a CNN for ACL injury detection on MRI images. Our model is based on ResNet and includes a dual attention mechanism to improve the performance of the model in diagnosing ACL injury. The proposed model was evaluated and compared with existing CNN models like MobileNet, EfficientNet-B0, EfficientNet-B1, VGG, ResNet-34, and ResNet-50.

## Methods

Patients diagnosed with an ACL injury from 2012 to 2020 were recruited from the Department of Minimally Invasive Surgery and Sports Medicine of The Second Affiliated Hospital of Harbin Medical University. Of the 400 cases considered for study inclusion, 10 cases were excluded after ACL reconstruction, 27 cases were excluded for severe OA, and 50 cases were excluded owing to a poor signal-to-noise ratio or motion artifacts. Finally, 313 patients aged 16–65 were included, including the MRI data of 368 subjects with an ACL injury and 100 subjects with intact ACL injury. The baseline characteristics of the population are shown in Table 1. All ACL diagnoses were confirmed by arthroscopic pathology and used as a reference standard for diagnosis.

**Table 1:** Baseline characteristics of the population (*: Mean ± Standard Deviation)

Items	results
Numbers	313 patients
	Injured ACL-220 patients(70.29%); Intact ACL-93 patients(29.71%)
Age(y)*	38.54 ± 12.14
Female/Male	131(41.85%) /182(58.15%)
BMI(kg/m2)*	24.55 ± 3.79
Selected Side	162-Left(51.76%)/151-Right(48.24%)

Inclusion criteria: Patients diagnosed with an ACL injury and underwent ACL reconstruction in hospital records.

Exclusion criteria: (1) Failing to find damaged ACL images in MRI; (2) Loss of imaging data of patients; (3) The patient was diagnosed with ACL injury before the operation, but the ACL was not treated during the operation, that is, no severe ACL injury was observed under arthroscopy; (4) The patient is younger than 18 years and older than 65 years.

**Fig. 1:**
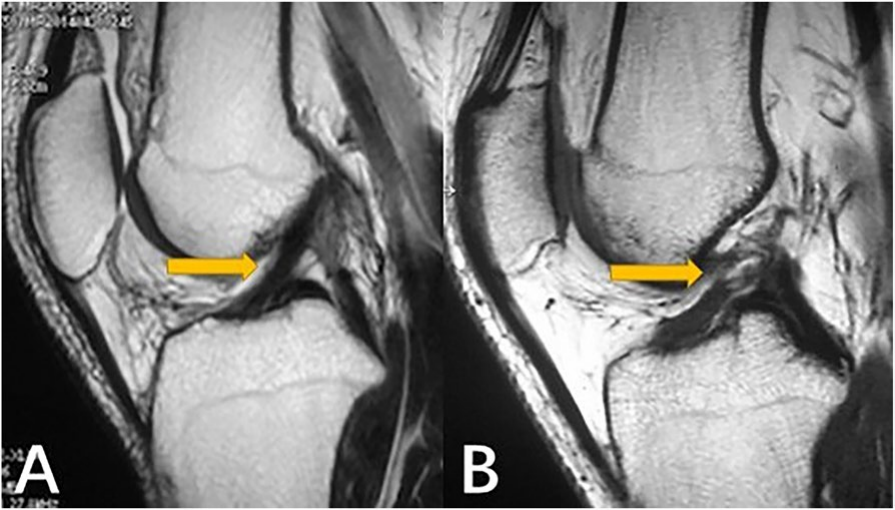
Yellow arrow: **A** Label 0-the intact ACL; **B** Label 1-the injured ACL

The MRI data of all the patients included in the study were selected and labeled by three orthopedic doctors. First, the layers with ACL images were selected in the sagittal MRI sequence of the knee joint to be cut into a single case of data. Second, three orthopedic surgeons marked whether the ACL was injured according to the images (Label 0 or Label 1, Fig. 1). Finally, the accuracy of the labeling results was determined by an orthopedic expert. A total of 468 knees sagittal MRI T1 sequence data were obtained, of which 100 were healthy, and 368 had an ACL injury, accompanied by the specified ACL diagnosis. The auxiliary diagnosis results outputted by our model were classified as Label 0 (Intact ACL) or Label 1 (Injured ACL).

**Fig. 2:**
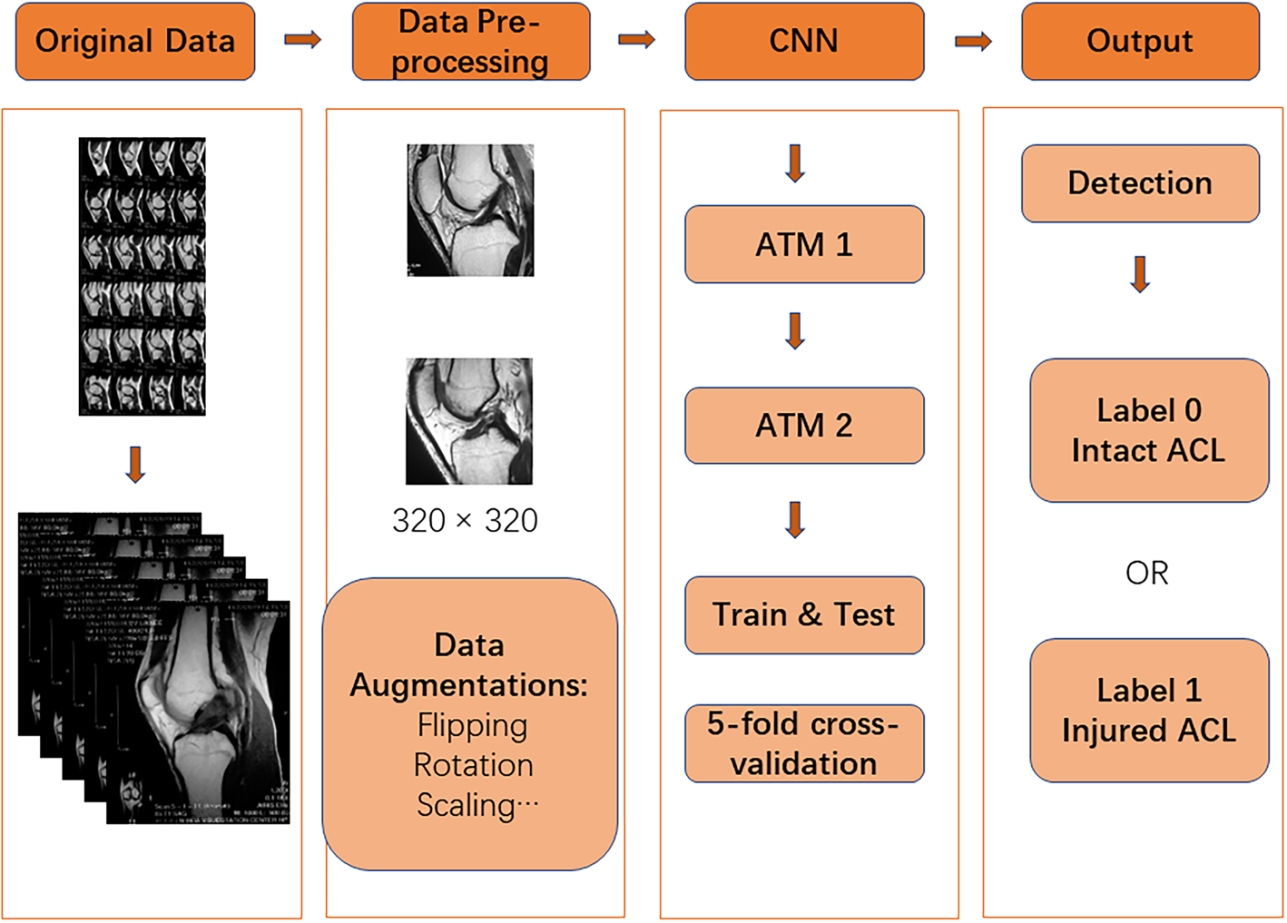
Flow chart of the proposed CNN model. The four stages include “Original Data”, “Data Pre-processing”, “CNN block” and “Output”

First, the length and width of the original data varied, so in the image preprocessing, we normalized all the original data, and the image size was 320 × 320. Second, we obtained a total of 468 images, of which 368 pieces were labeled “1,” and 100 pieces were labeled “0.” As few pieces were Label 0, we needed to solve the data imbalance problem to prevent underfitting in our trained model. By adding flipping, rotation, scaling, and other methods to expand the data, we expanded the dataset Label 0 to 275 pieces. All data were reviewed by a chief orthopedic physician before being processed through machine learning, with the same exclusion criteria as above, and then 13 pieces of Label 1 that did not meet the criteria were removed. The final data set was 630 pieces, including 355 pieces of Label 1 and 275 pieces of Label 0. The block diagram of our model's operation procedure is shown in Fig. 2, including four main stages.

In the data input stage, the patient's knee MRI images are cut into a single piece of data. In the second stage, the data pre-processing stage, all single data are normalized, and the data augmentations are expanded by flipping, rotating, scaling and other methods [13]. The CNN model recognition stage is completed by our proposed CNN model. On the base of ResNet, two different attention mechanism modules are added that enhance the recognition ability of the model and effectively improve the accuracy of the model to identify ACL injuries. Then the performance is measured and compared by random five-fold cross-validation. Finally, the identification results of the CNN model (Label 0 or Label 1) are output by detecting the ACL injury.

**Fig. 3:**
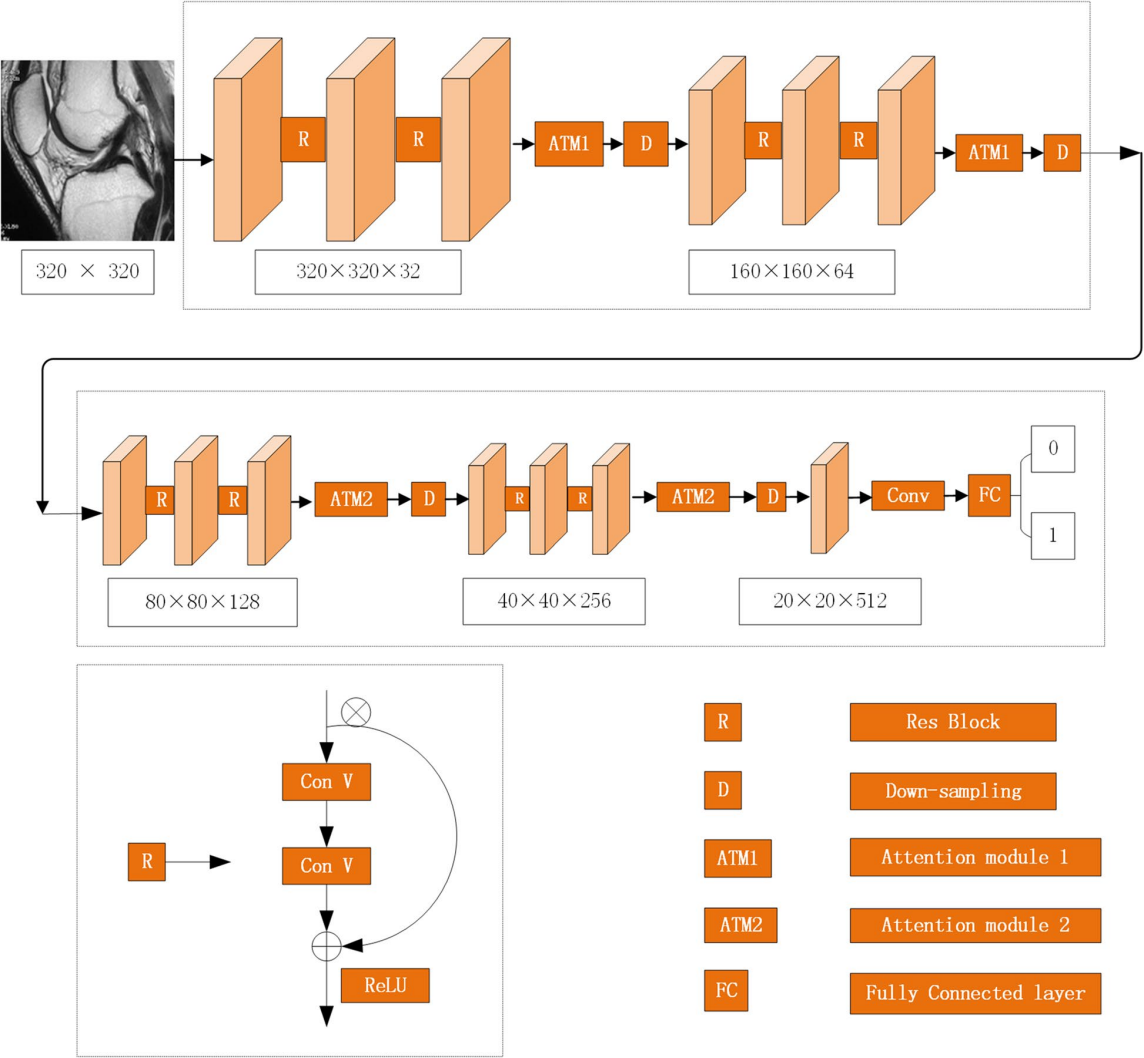
Structure of our convolutional neural network model. The CNN model proposed in this paper is roughly divided into two parts. The first part is mainly supported by ATM1, which enhances feature extraction. The latter part is mainly supported by ATM2 to enhance the classification. The lower left is the structure of the ResNet, and the “ReLU” is the activation function of the ResNet. The method used for down-sampling was max pooling with a step size of 2

The proposed CNN model is based on a 13-layer ResNet [14] and adopts two attention mechanism modules which were inspired by previous research (ATM1 [15] and ATM2 [16]) to improve the accuracy of model recognition, as shown in Fig. 3. General, the attention mechanism is mainly applied to the processing of the middle layer feature map of the neural networks, which aims to improve the ability of feature extraction to achieve better identification.

The utility of the attention mechanism (ATM) in neural networks for image processing has been widely noticed by scholars, and several classical ATM networks exist, such as SENet [17] and ECA [16]. The ATM’s working principle can be considered as simulating the human visual attention mechanism; by scanning the entire target image, focusing on the target region, and then devoting more resources to this region to obtain key information. Through the ATM, limited attention resources can be used to quickly identify key information from a large amount of information, reduce attention to other information, and even filter out irrelevant information. The mechanism can therefore improve the efficiency and accuracy of image processing. In medical imaging, the ATM can help neural networks focus on key information when processing large amounts of medical image data. As a result, the ATM has been applied to the medical field, including in assisting clinicians with the identification of melanoma [18], retinal lesions [19], the pathological sections of colorectal cancer [20] and breast cancer [21], and it has achieved excellent results. Thus, we believe that adding ATM to our CNN model can effectively improve the recognition efficiency and accuracy of the model for knee MRI images.

In short, when the ATM was computing, the input medical image feature was defined as Q(queries), K(keys), and V(values). The ATM was to calculate the attention weight between Q and K, and then enhanced V, which Q, K, and V came from the same medical image feature. The formula for ATM was given in (1) [22], which computed the attention function on a set of queries simultaneously, packed together into a matrix Q. The keys and values were also packed together into matrices K and V. So essentially ATM was a weighted sum of the values of the elements in the source, and Q and K are used to compute the weight coefficients of the corresponding values. Then ATM computed the dot products of the Q with all K, divided each by $\sqrt{{\mathrm{d}}_{\mathrm{k}}}$, and applied a Softmax function to obtain the weights on the values. Thus, it simulated the human visual attention mechanism, devoting more resources to key information and less attention to other information.

1 $$Attention\left(Q, K, V\right)=softmax\left(\frac{{QK}^{T}}{\sqrt{{\mathrm{d}}_{\mathrm{k}}}}\right)V$$

**Fig. 4:**
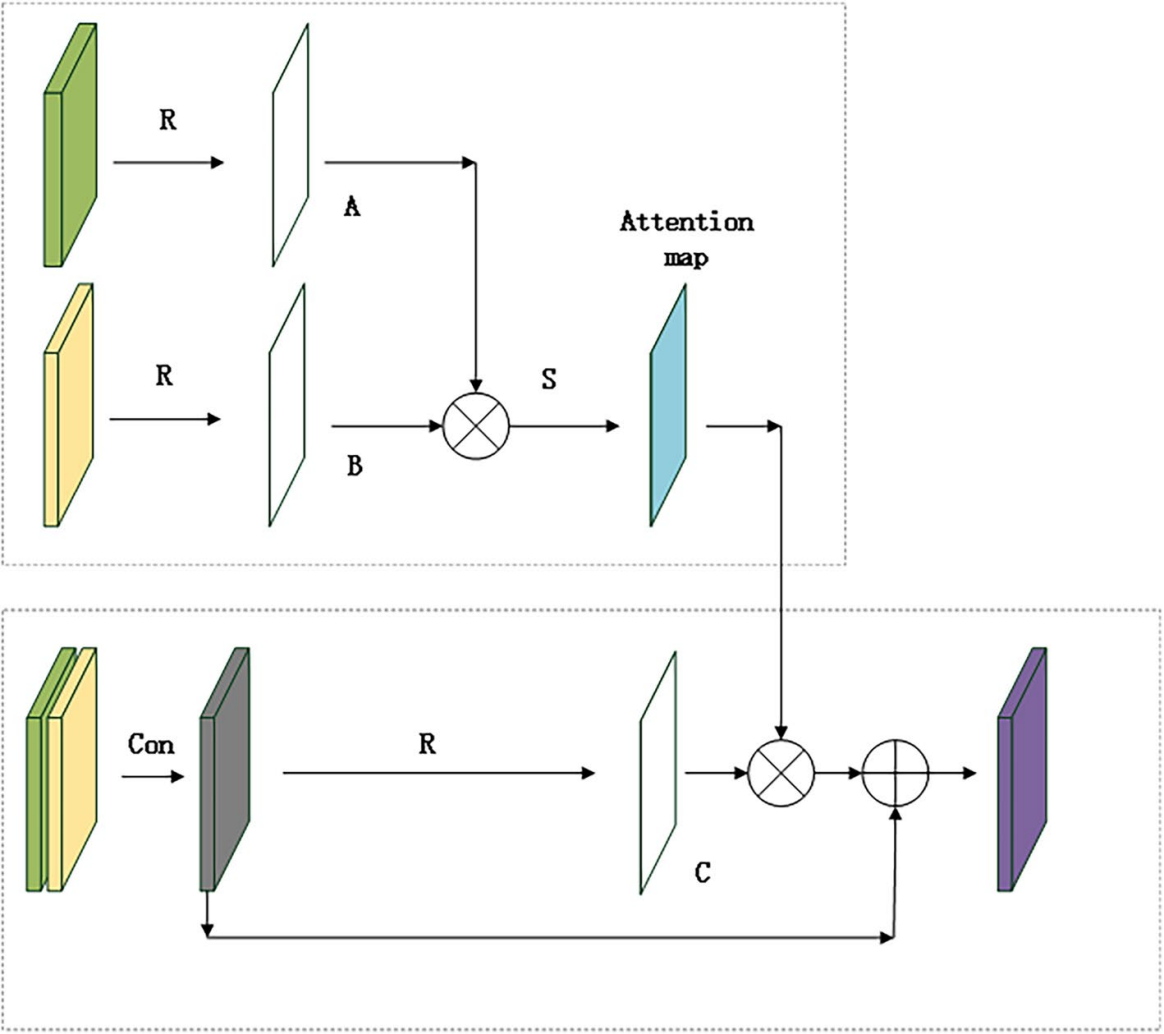
Structure of attention mechanism module 1(ATM1). Green block and yellow block are the symmetrical features in the encoding and decoding process. To reduce the dimension of the channel, a convolutional layer is connected after the green block and the yellow block, obtaining the grey block. Purple block is the finally result of enhanced feature extraction. R denotes reshape, S denotes softmax activation function, □denotes matrix multiplication, ⊕denotes matrix addition

ATM1 is mainly used in the primary stage of the network. At this time, the feature size is large, and the number of channels is small, so ATM1 is mainly used to capture the lesion part of the image’s spatial characteristics. Therefore, a spatial attention module is designed in the front of the network. In image segmentation, ATM1 enhances the ability of the network to extract image features through three stages: feature extraction, feature similarity calculation, and original feature enhancement. The attention module aims to extract similar features in the CNN; and then uses similarity to enhance the original features. In short, the function of ATM1 is to map the original features of data to three spaces: A, B, and C. Because the features in the A, B, and C spaces roughly follow the same distribution, the features’ similarity in the A and B spaces can enhance the features in the C space, improving the feature extraction ability and enhancing the ability of original features. The structure of the ATM1 module is shown in Fig. 4.

**Fig. 5:**
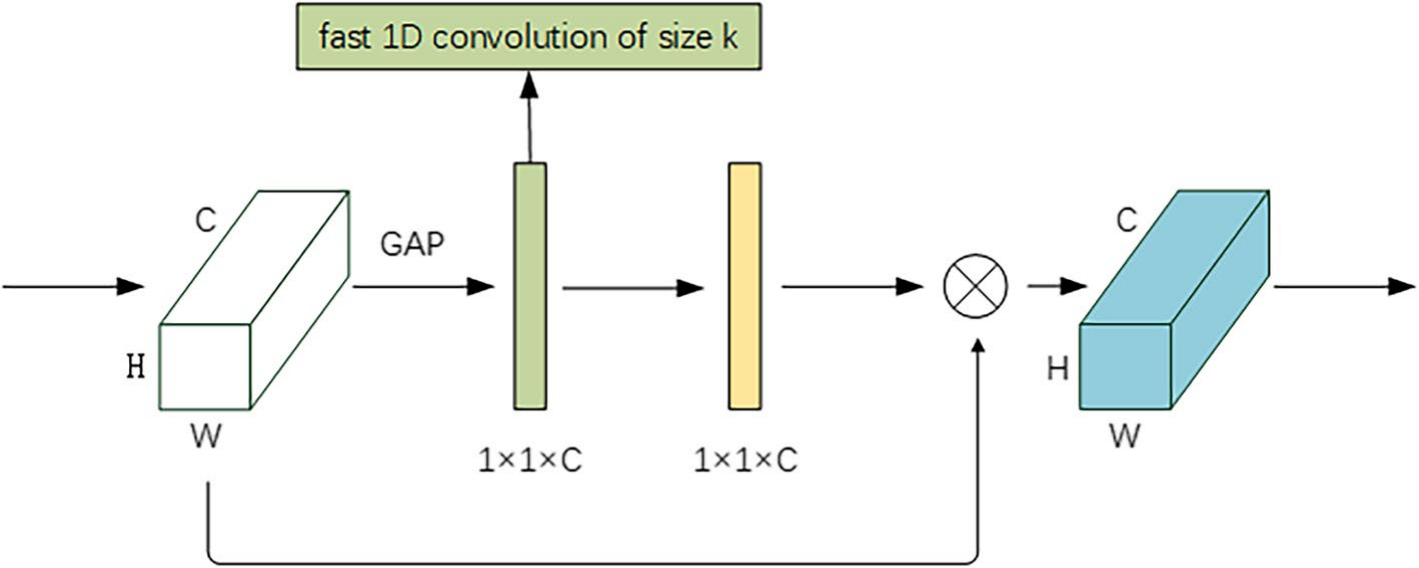
Diagram of attention mechanism module 2(ATM2). The white block is the feature obtained by convolution which height is H, width is W, channel dimension is C), and the green block’s size is 1 × 1 × C. In this block, ATM2 carries out fast 1D convolution with size k (k is adaptive selected with the channel dimension C), and the result is the yellow block. Then it is calculated with the features before the ATM2 calculation, gaining the finally result which is the blue block

ATM2 draws on an efficient channel attention (ECA) module. Because involves fewer parameters, it reduces the computational complexity of the neural network model and produces a clear performance gain. In our CNN proposed model, ATM2 is mainly used in the late stage of the network. In this stage, the image features are mainly transformed into abstract high-level features, and these extracted features are used to map to the lesion category. Therefore, it is necessary to strengthen the mapping function in the back of the network. A channel attention module is designed to strengthen the classification ability of the model to extract features. The diagram of the ATM2 module is shown in Fig. 5.

Overall, the ATM1 was a spatial attention mechanism and the ATM2 was a channel attention mechanism, where the former further enhanced the features through the spatial similarity of the extracted features, while the latter focused on the classification ability of feature extraction. Our proposed ATM1 + ATM2, on the other hand, joined ATM1 and ATM2 into the whole network to fully utilize the respective features of the two attention mechanisms in order to enhance the effectiveness of the whole network. However, previous studies only added one type of ATM to the CNN model, so our research focused on adding two different ATM modules to the CNN model to verify whether it could improve the performance of the CNN model and whether it performed better than one ATM alone by using the mechanism of different ATMs. Therefore, we were inspired by existing studies and choose two ATMs to add to our proposed CNN model.

## Results

**Fig. 6:**
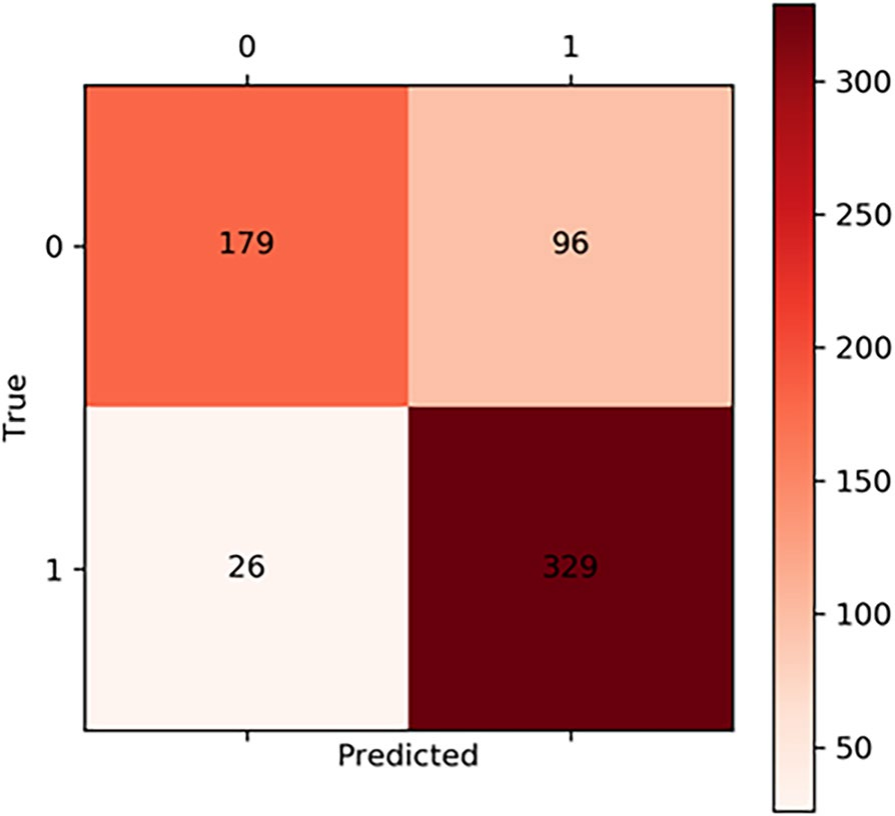
Confusion matrix of our CNN model testing all data

**Fig. 7:**
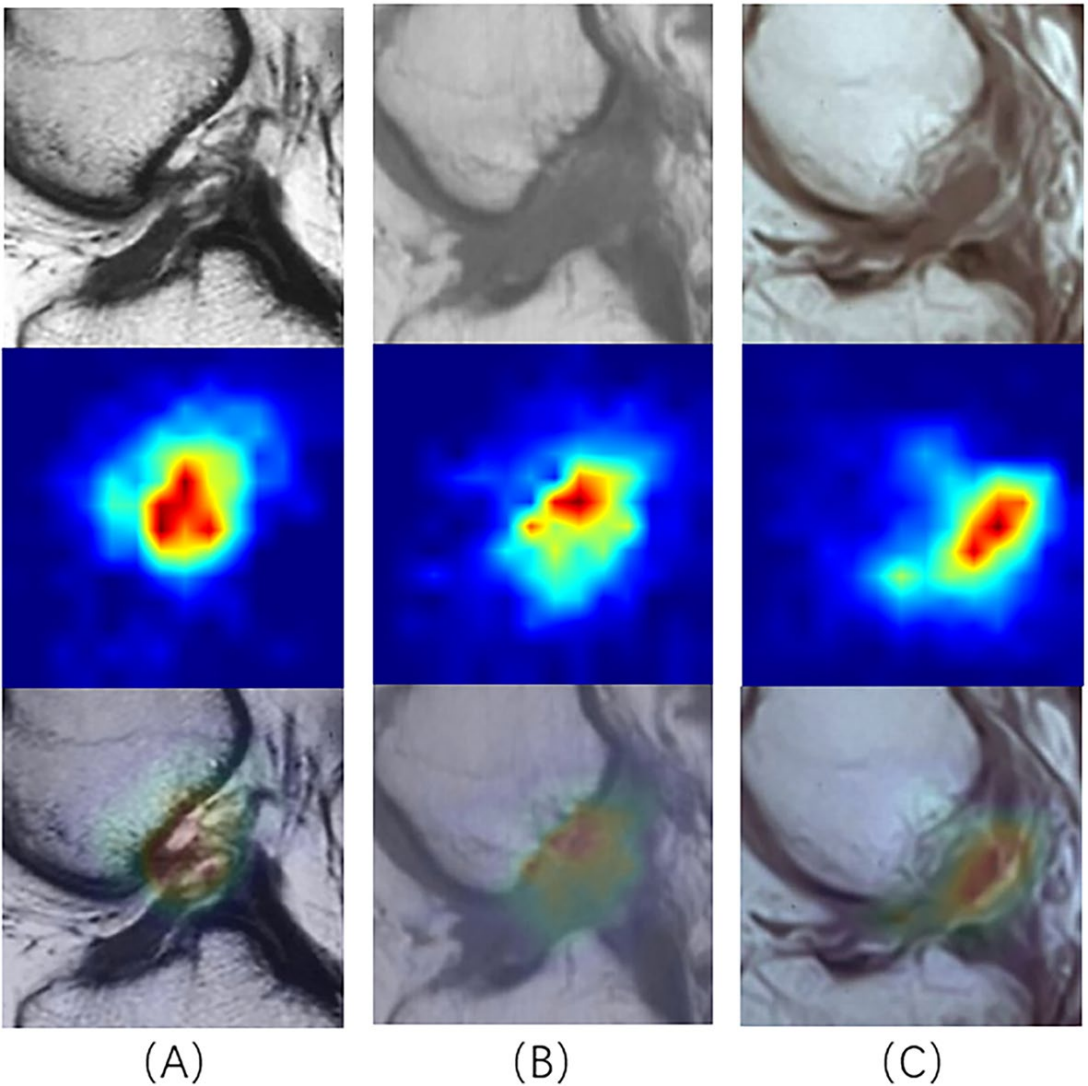
Top: Input data with injured ACL; Middle: the areas that may have injured ACL identified by the model, and the “Hotter” areas indicate the existence of injured ACL; Bottom: Visualization of model identification results on input data

Our model with the dual attention mechanism was tested on 630 training data sets and achieved the following performance: accuracy: 0.8063, precision: 0.7741, sensitivity: 0.9268, specificity: 0.6509, the area under the curve (AUC): 0.8886, and F1-score: 0.8436. Meanwhile, our model had lighter Parameters than other models. The evaluation results of our model compared with those of MobileNet, EfficientNet-B0, EfficientNet-B1, VGG, ResNet-34, and ResNet-50 are shown in Table 2. Moreover, the results of ATM1 and ATM2 show that one attention mechanism in the network structure was based on our CNN model. The results show that our model combining ATM1 and ATM2 in the neural network performed better, and that although the accuracy of ATM1 was higher than the combination, its AUC, sensitivity, and F1 scores were lower. Thus, overall, the combination of ATM1 and ATM2 performed better than ATM1 alone. We also showed the results compared with existing attention mechanisms in Table 3. Also, our model had better performance than SENet and CBAM. The confusion matrix of all data is shown in Fig. 6, which shows the true positive, true negative, false positive, and false negative of Label 0 and Label 1. Our model visualization of the identification of ACL injuries on input data is shown in Fig. 7.

**Table 2:** Evaluation of CNN Models. Our model performed well in accuracy, sensitivity, AUC, F1-score and had lighter parameters

Model	Accuracy	Precision	**Sensitivity**	Specificity	AUC	F1-score	**Parameters**
MobileNet	0.6397	0.6975	0.6366	0.6036	0.8335	0.7979	5.35M
EfficientNet-B0	0.7413	0.7500	0.8113	0.6059	0.7820	0.7794	5.22M
EfficientNet-B1	0.7460	0.7481	0.8282	0.6400	0.8126	0.7861	7.72M
VGG	0.7619	0.8213	0.7380	0.7927	0.8523	0.7774	15.29M
ResNet-34	0.7524	0.7386	0.8676	0.6036	0.8335	0.7979	22.36M
ResNet-50	0.7889	0.7921	0.8479	0.7127	0.8627	0.8190	25.32M
**Ours**	**0.8063**	0.7741	**0.9268**	0.6509	**0.8886**	**0.8436**	**2.23M**

**Table 3:** Comparison of ATMs. Compared with SENet and CBAM, our model had better performance in accuracy, sensitivity, AUC, and F1-score

Model	Accuracy	Precision	**Sensitivity**	Specificity	AUC	F1-score
SENet	0.7905	0.8005	0.8366	0.7309	0.8646	0.8128
CBAM	0.8127	0.8496	0.8113	0.8145	0.8842	0.8300
ATM1	0.8111	0.8041	0.8789	0.7236	0.8786	0.8398
ATM2	0.7667	0.8824	0.6761	0.8836	0.8831	0.7656
**Ours**	**0.8063**	0.7741	**0.9268**	0.6509	**0.8886**	**0.8436**

**Fig. 8:**
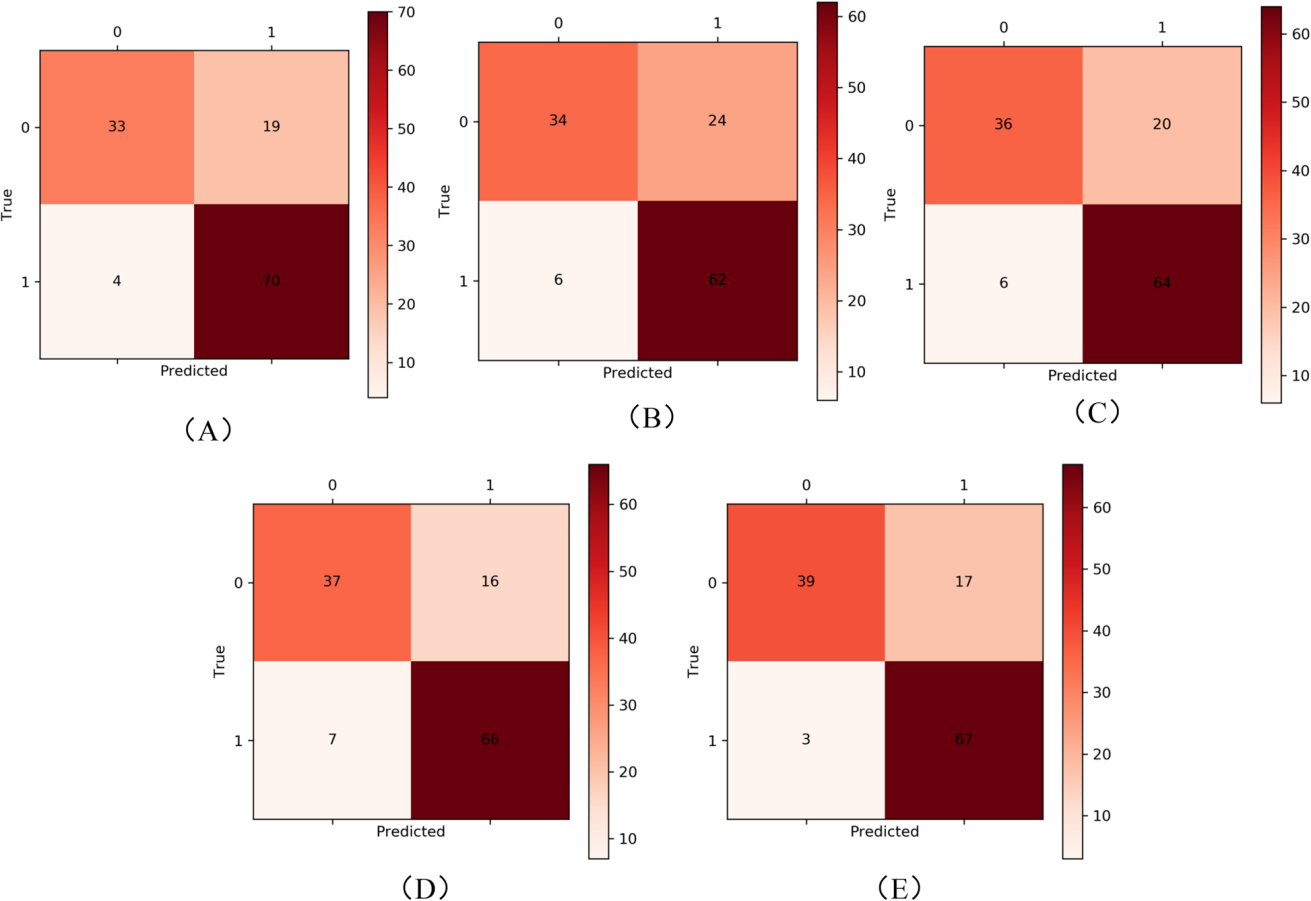
Confusion matrix of fivefold cross-validation. **A** Acc = 0.8175; **B** Acc = 0.7619; **C** Acc = 0.7937; **D** Acc = 0.8175; **E** Acc = 0.841

In the five-fold cross-validation, 80% of the dataset was randomly selected for training each time, and 20% of the remaining dataset was used as the validation set. The confusion matrix of five-fold cross-validation is shown in Fig. 8(A-E). The average accuracy in the five-fold cross-validation was 0.8064.

**Fig. 9:**
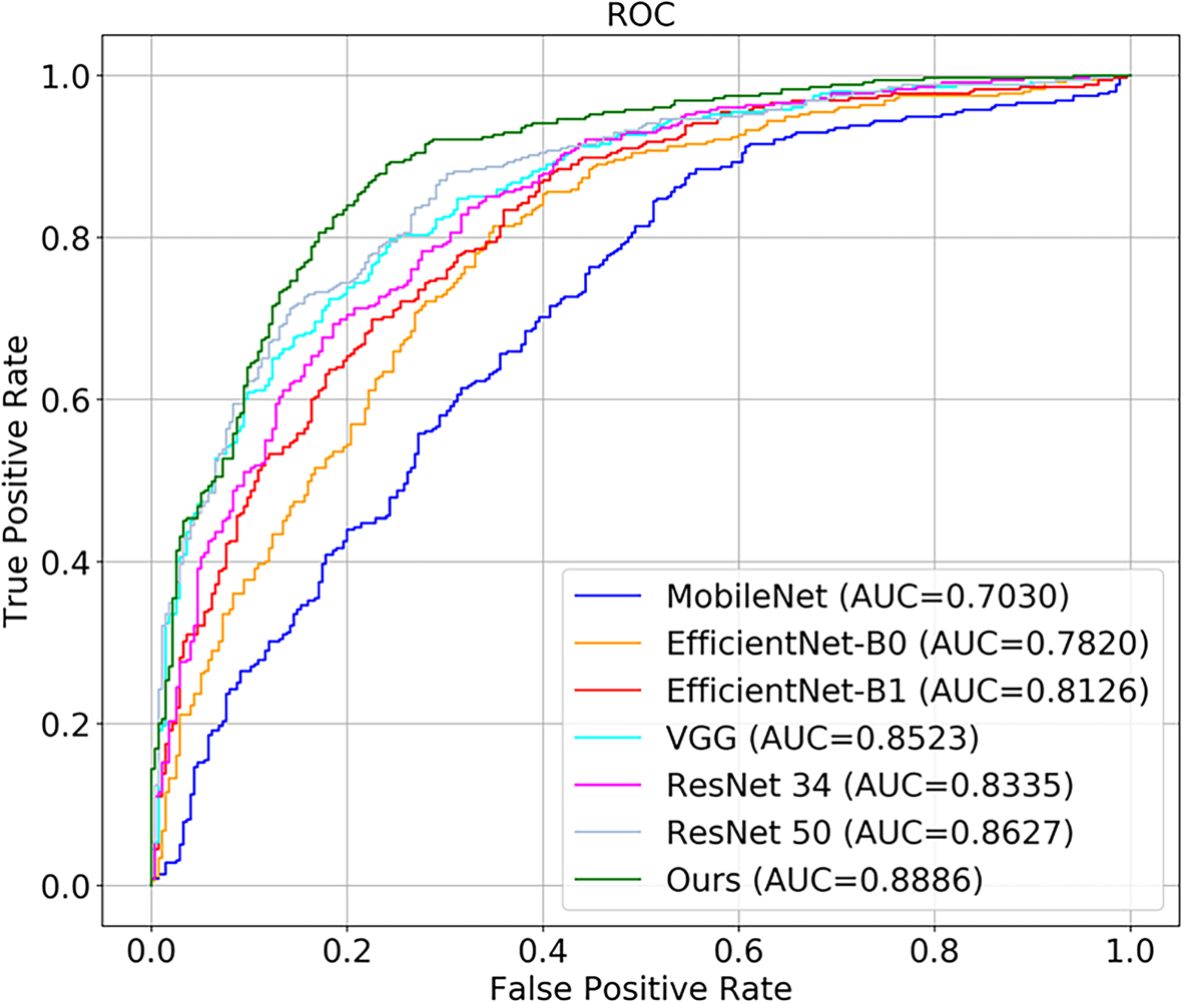
The area under curves of our Model compared with other CNN models

**Fig. 10:**
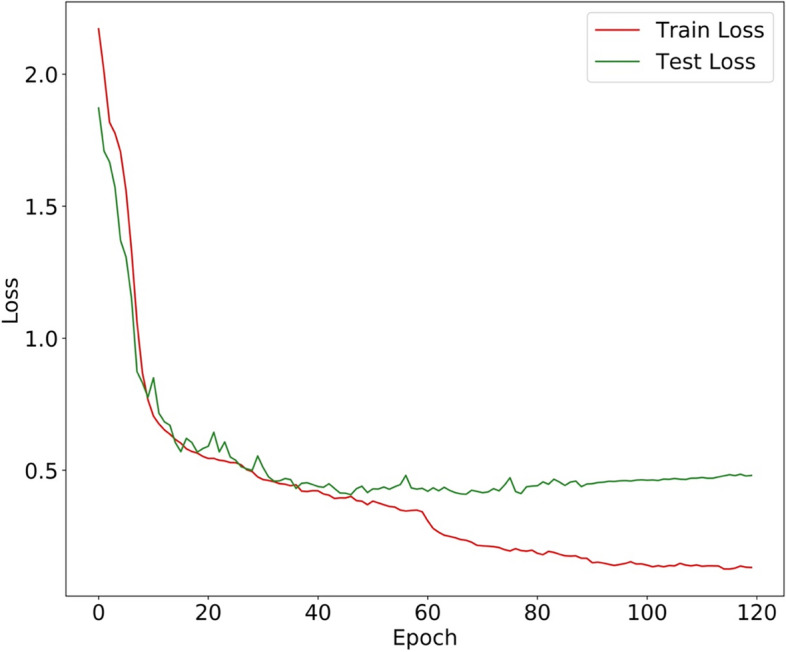
The loss graph of training and testing dataset

Figure 9 shows the receiver operating characteristic curve (ROC curve); of all the CNN models, our model achieved the highest AUC (0.8886), and it outperformed MobileNet (0.7030), EfficientNet-B0 (0.7820), EfficientNet-B1 (0.8126), VGG (0.8523), ResNet-34 (0.8335), and ResNet-50 (0.8627). Meanwhile the loss graph of training and testing dataset was shown in Fig. 10, which told that test-loss has stabilized.

## Discussion

**Fig. 11:**
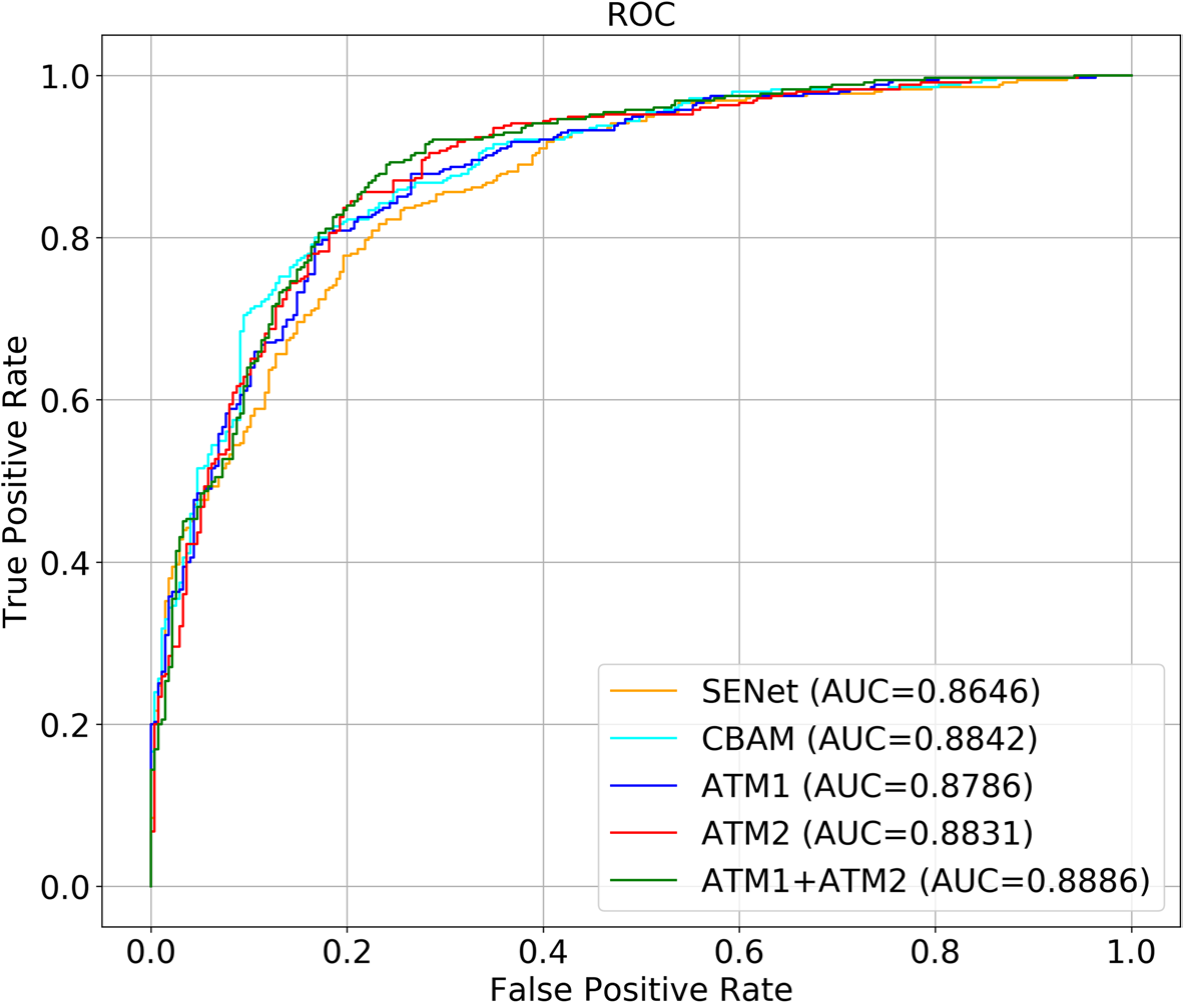
The area under curves of our Model compared with SENet, CBAM, and only use ATM1 or ATM2

In this study, we demonstrated, in detail, a CNN model for automatic ACL detection. We studied the problem using 13 layers of a CNN custom residual network based on deep learning to effectively detect ACL damage. We also compared the performance of our model using two different attention mechanisms against that of ATM1 and ATM2 alone. As shown in Fig. 11, the performance of models with ATM1 and ATM2 alone were similar to that of combined applications. This result shows that ATM1 and ATM2 were beneficial to the model, in which the AUC (ATM1 + ATM2) is better than AUC (ATM2) and AUC (ATM1), as shown in Fig. 11. The average accuracy of five-fold cross-validation was 0.8064.

**Fig. 12:**
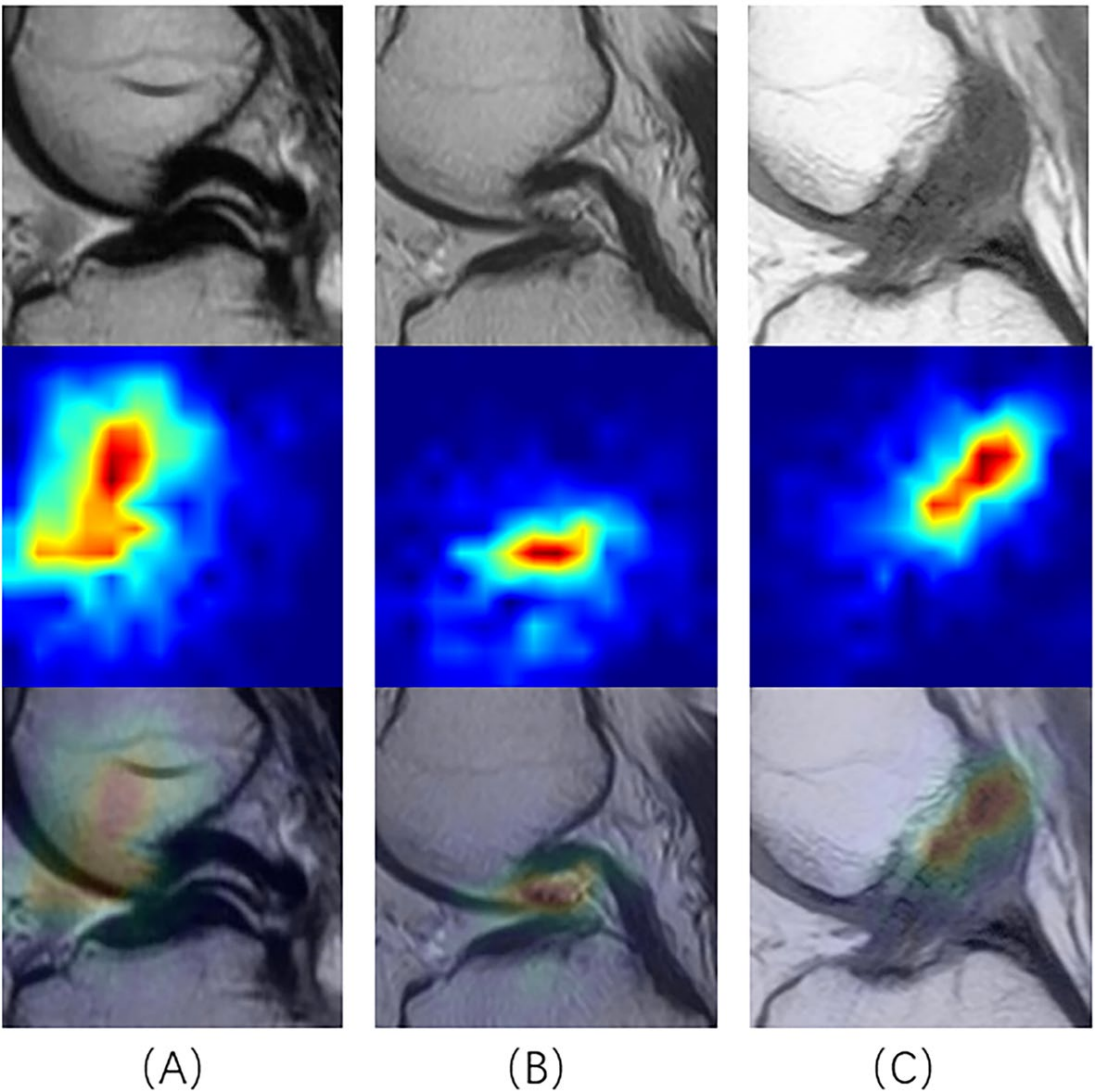
Abnormal identification of damaged area by our model. Top: Input data with injured ACL; Middle: the areas that may have injured ACL identified by the model, and the “Hotter” areas indicate the existence of injured ACL; Bottom: Visualization of model identification results on input data. But the damage area identified by the model is not the area of injured ACL

At the same time, there were some limitations in our research: (1) Complete damage and partial damage could not be distinguished, and the fine features of the model on the image still need to be improved; (2) For patients with obsolete injuries, if the ACL had been absorbed such that there was no ACL in the MRI image of the patient, the model would identify it as Label 0; (3) We did not have much model training data, and we lacked external data validation; (4) Anomaly identification appeared in the thermal map of model identification whether ACL had been damaged or not, as shown in Fig. 12.

Subsequently, we randomly selected 50 cases of data in the original data set, including 37 cases of Label 1 and 13 cases of Label 0. We asked two orthopedic residents, one orthopedic chief physician, two radiology residents, and one radiology resident in our hospital, all of whom had been blinded to the experiment, to annotate these 50 cases of data. One of the two residents annotated directly, and the other one annotated the diagnosis results with the aid of our CNN model. At the time of the resident's diagnosis, in addition to the MRI images, we also provided a heat map of these images after they had been identified by our CNN model, as in the results in Fig. 7. This approach assisted the resident in the diagnosis. Then, the diagnostic accuracy and sensitivity were calculated. The results are shown in Table 4. Although the accuracy and sensitivity of the residents with the assistance of the CNN model were much lower than those of the chief physicians, the CNN model still improved the diagnostic accuracy of residents for ACL injuries. In addition, it did not take much time for the CNN model to identify data, which can greatly accelerate the diagnostic efficiency of clinicians. Inevitably, the accuracy and specificity of our proposed model for identifying ACL injuries were far from that of experienced chief orthopedic surgeons and chief radiologists. We believed that the possible reasons for this include: (1) the sample size of this randomly selected test was slightly small, which lead to a large gap in the results; (2) although the efficacy of the model was good and the test loss shown in Fig. 10 was stabilized, the number of training sets was not sufficiently large and there was no validation of the external data. So, our research direction was not only to further optimize the structure of the network with the dual-attention mechanism but also to collect more training data sets and external data to improve the efficacy of the network.

**Table 4:** The result of the selected data. Residents gained well outcomes with the aiding of CNN model. The diagnostic result of randomly selected data set

Operator	Accuracy	Sensitivity
Chief orthopedic physician	0.98	0.97
Orthopedic resident 1	0.74	0.70
Orthopedic resident 2 with CNN	0.82	0.81
Chief Radiologist	0.96	0.97
Radiology resident 1	0.76	0.73
Radiology resident 2 with CNN	0.84	0.84

In existing studies using CNN to assist clinicians in the diagnosis of ACL injuries, Štajduhar et al. [23]. obtained a highly effective model by training it on 917 data sets in 2017. A classic study used Bien et al.'s MRNet [24], using 1370 cases of data in which the accuracy of an ACL injury was 0.867, the sensitivity was 0.759, and the AUC was 0.965. However, when Štajduhar et al.'s 917 data sets were used as external data for validation, the efficiency of the model decreased. Therefore, the external data validation of the CNN model was highly important, and external validation was needed to test the generalization ability of the model. Irmakci et al. [25] also used Bien's data set to train three different CNN models, and they concluded that ResNet-18 had better performance. Furthermore, Tsai et al. [26] proposed a new model. On the same data set, the model's performance was better than that of MRNet, and they achieved good results on the external data set of Štajduhar et al. Awan et al. [27] also used the data set training of Štajduhar et al. to identify an intact ACL, partially torn ACL and ruptured ACL based on the ResNet model, and they achieved the best results thus far (intact ACL: accuracy = 0.92, AUC = 0.98; partial tear ACL: accuracy = 0.91, AUC = 0.97; ruptured ACL: accuracy = 0.93, AUC = 0.99). Other studies used similar methods to customize novel CNN models, and they used classical data sets or data collected by researchers to test the auxiliary diagnostic efficiency of the model. They achieved good outcomes, proving that a CNN can be used as an efficient tool to help clinicians detect ACL injuries.

With the iteration of the CNN model, it is bound to be updated, and more efficient models will be used for training and learning. This means that the existing research results will always be covered by iterative models and algorithms, which cannot achieve the purpose of transforming research results and providing more help to clinicians. Additionally, the data set contains limitations. In the existing research, the original data set of CNN learning, training and verification are mostly the same. The issue with using different CNN models to train and analyze the same data is that new model are generally superior to older ones, casting doubt on the generalization ability of the model. That is, when a CNN model analyzes unknown data, its efficiency may not be as good as when learning and training known data, so the external verification of the model is particularly important.

In sum, the theories of using a CNN as a clinical decision support system to assist clinicians in the diagnosis of ACL injury have been mature. In the continuous updating of the CNN model, a CNN can also achieve better accuracy and sensitivity. Moreover, the input data of the model are no longer limited to similar data sets, and the classification criteria are no longer divided into whether the ACL is injured, or uninjured, so we must turn to the prediction of ACL injuries [28] and ACL reconstruction failures. However, the process of transforming clinical trial result and applying this technology in a such a way that helps clinicians remains stagnant. Therefore, future research should focus on interdisciplinary medicine and engineering. Researchers should speed up the transformation of clinical trial results, so that CNN models, as a clinical decision support system, can be used by clinicians as soon as possible, reducing the misdiagnosis rate of ACL injuries and increasing clinician’s diagnostic efficiency.

## Conclusion

In this paper, an effective automatic CNN model was proposed to detect ACL injuries from MRI images of the knees. The model is based on ResNet and uses a dual attention mechanism to identify an ACL injury in an MRI image. We obtained an AUC of 0.8886 and performed testing by five-fold cross-validation. The result indicated that the model can effectively help orthopedic clinicians and radiologists diagnose ACL injuries, improve diagnostic efficiency, and reduce misdiagnosis and missed diagnoses.

## Data Availability

The datasets used and analyzed during the current study are available from the corresponding author on reasonable request.

## Abbreviations

ACL: Anterior cruciate ligament
ATM: Attention mechanism
MRI: Magnetic resonance imaging
CNN: Convolutional neural network
AUC: Average area under curves
ROC curve: Receiver operating characteristic curve
